# CLL stereotyped B-cell receptor immunoglobulin sequences are recurrent in the B-cell repertoire of healthy individuals: Apparent lack of central and early peripheral tolerance censoring

**DOI:** 10.3389/fonc.2023.1112879

**Published:** 2023-03-17

**Authors:** Stefano Vergani, Davide Bagnara, Andreas Agathangelidis, Anita Kar Yun Ng, Gerardo Ferrer, Andrea N. Mazzarello, Florencia Palacios, Sophia Yancopoulos, Xiao-Jie Yan, Jaqueline C. Barrientos, Kanti R. Rai, Kostas Stamatopoulos, Nicholas Chiorazzi

**Affiliations:** ^1^ Karches Center for Oncology Research, The Feinstein Institutes for Medical Research, Northwell Health, Manhasset, NY, United States; ^2^ Centre for Research and Technology Hellas, Institute of Applied Biosciences, Thessaloniki, Greece; ^3^ Department of Biology, School of Science, National and Kapodistrian University of Athens, Athens, Greece; ^4^ New York Genome Center, New York, NY, United States

**Keywords:** CLL (chronic lymphocytic leukemia), B cell development and differentiation, B cell repertoire, stereotyped antigen receptors, VDJ sequencing

## Abstract

**Introduction:**

The leukemic cells of patients with chronic lymphocytic leukemia (CLL) are often unique, expressing remarkably similar IGHV-IGHD-IGHJ gene rearrangements, “stereotyped BCRs”. The B-cell receptors (BCRs) on CLL cells are also distinctive in often deriving from autoreactive B lymphocytes, leading to the assumption of a defect in immune tolerance.

**Results:**

Using bulk and single-cell immunoglobulin heavy and light chain variable domain sequencing, we enumerated CLL stereotype-like IGHV-IGHD-IGHJ sequences (CLL-SLS) in B cells from cord blood (CB) and adult peripheral blood (PBMC) and bone marrow (BM of healthy donors. CLL-SLS were found at similar frequencies among CB, BM, and PBMC, suggesting that age does not influence CLL-SLS levels. Moreover, the frequencies of CLL-SLS did not differ among B lymphocytes in the BM at early stages of development, and only re-circulating marginal zone B cells contained significantly higher CLL-SLS frequencies than other mature B-cell subpopulations. Although we identified CLL-SLS corresponding to most of the CLL major stereotyped subsets, CLL-SLS frequencies did not correlate with those found in patients. Interestingly, in CB samples, half of the CLL-SLS identified were attributed to two IGHV-mutated subsets. We also found satellite CLL-SLS among the same normal samples, and they were also enriched in naïve B cells but unexpectedly, these were ~10-fold higher than standard CLL-SLS. In general, IGHV-mutated CLL-SLS subsets were enriched among antigen-experienced B-cell subpopulations, and IGHV-unmutated CLL-SLS were found mostly in antigen-inexperienced B cells. Nevertheless, CLL-SLS with an IGHV-mutation status matching that of CLL clones varied among the normal B-cell subpopulations, suggesting that specific CLL-SLS could originate from distinct subpopulations of normal B cells. Lastly, using single-cell DNA sequencing, we identified paired IGH and IGL rearrangements in normal B lymphocytes resembling those of stereotyped BCRs in CLL, although some differed from those in patients based on IG isotype or somatic mutation.

**Discussion:**

CLL-SLS are present in normal B-lymphocyte populations at all stages of development. Thus, despite their autoreactive profile they are not deleted by central tolerance mechanisms, possibly because the level of autoreactivity is not registered as dangerous by deletion mechanisms or because editing of L-chain variable genes occurred which our experimental approach could not identify.

## Introduction

Chronic lymphocytic leukemia (CLL) is a disease characterized by the expansion of a CD5^+^ B cell clone in the peripheral blood, bone marrow (BM), and secondary lymphoid tissues (1). The development of the disease strongly correlates with age, with a median age at diagnosis of ~70 years. The antigen receptor on the surface membrane of a B cell (BCR) plays a key role in the development and evolution of CLL as indicated by multiple studies (2–7). Most extraordinary among these studies is the remarkable similarity in the amino acid sequences of the antigen binding domains of the BCRs from certain CLL patients (8, 9). Analyses of large patient cohorts indicate that this is a recurrent feature in at least 40% of CLL clones (10). Indeed, patients can be divided into specific stereotyped subsets based on similarity in the VH CDR3 of the IGHV-IGHD-IGHJ (IGHV-D-J) rearrangement, and patients bearing discrete stereotyped BCRs can have unique clinical features and outcomes and have leukemic clones with distinct specific genomic aberrations (11, 12).

Based on the structural distinctiveness and clinical importance of BCRs in the disease, recombinant CLL IGs have been studied for antigen reactivity, revealing binding to a variety of exo- and auto-antigens (13–17). Documentation that reversion to the germline IGHV sequence converted certain exo-reactive to auto-reactive IGs (13, 14) led to the notion that CLL derives from an autoreactive B lymphocyte.

Because of the potentially harmful capacities of autoreactive clones for healthy people, evolutionarily a series of immunologic censoring mechanisms have evolved to eliminate or to reduce the avidity of autoreactive B cells during the early phase of development (18). In this regard, the existence of apparently “CLL-specific IGHV-D-J rearrangements” in the healthy B-cell repertoire is an important but relatively unstudied issue as current information is only available for mature circulating and splenic B cells (19–22). Whether such B cells from normal individuals, which would be expected to be self-reactive, are subjected to immunologic censoring mechanisms during development is not known.

Using a sensitive IGHV-D-J deep-sequencing approach (23), we sought to identify stereotyped IGHV-D-J rearrangements in B lymphocytes from healthy people at various stages of B-cell maturation In particular, considering the auto-reactive nature of CLL IGs, we set out to determine at which checkpoints such stereotyped rearrangements were triaged from the B-cell repertoires of normal individuals to maintain immune tolerance.

Our studies indicate that IGHV-D-J gene sequences resembling stereotyped CLL BCRs and belonging to one of the 29 major CLL stereotyped subsets are present in the normal B-cell repertoire. They are found at different sites, such as cord blood (CB), BM, and peripheral blood. Despite their autoreactive features, they do not appear to be purged during early B-cell development, the first checkpoints to sustain immunologic tolerance in the healthy setting.

## Material and methods

### Samples

The study was approved by the Institutional Review Board of Northwell Health. Bone marrow (BM) samples were collected as discarded bone segments from anonymized patients who had undergone joint replacement surgery. Persons with a history of any autoimmune disease or condition and of any cancer were excluded from the study. Peripheral blood and umbilical cord blood samples were similarly collected from anonymous healthy donors. Mononuclear cell (MC) fractions were separated by density gradient centrifugation (Ficoll, GE Healthcare), frozen (10% DMSO 45% FBS and 45% RPMI1640) and stored in liquid nitrogen until used.

### Processing of BM samples

BM samples were placed in a large Petri dish containing cold PBS with 2.5% BSA, and the tissue was gently dissociated using the plunger of a 60 ml sterile plastic syringe. Bone fragments were broken into small pieces using scissors and rinsed with the same buffer to extract cells from tissue niches. Cell suspensions were then passed through a 70uM cell strainer into a 50 ml tube. To optimize the yield, after processing Petri dishes were rinsed with buffer used for the dissociation, and the contents were added to the previously filtered suspension. BMMCs were separated by Ficoll density gradient centrifugation, frozen (10% DMSO 90%FBS), and stored in liquid nitrogen until used.

### Isolation of various B-cell subpopulations by cell sorting

BM cell suspensions were incubated with V500 anti-CD19 and with PE-cy7 anti-CD10 mAbs (both BD Biosciences) for 20 minutes at 4°C, and after washing were sorted into CD10^+^ and CD10^-^ fractions. Non-B cells were excluded by using efluor-450 anti-CD3 and anti-CD16 mAbs, and dead cells were triaged by Sytox Blue (ThermoFisher) staining. The CD10^+^ fractions were then additionally stained with FITC anti-CD34 (BD Biosciences), PE anti-IgM (Ebioscience) and eflour-450 anti-CD27 (Ebioscience) to further prohibit contamination with mature B cells. Pro B cells (PRO, CD34^+^IgM^-^), Pre B cells (PRE, CD34^-^IgM^-^) and immature B cells (IMM, CD34^-^IgM^+^) were collected.

CD10^-^ fractions were also stained with PerCPcy5.5 anti-CD38 (BioLegend), FITC anti-IgD (ThermoFisher), APC anti-CD27 (BD Bioscience), and PE anti-CD24 (Bioscience) to discriminate naïve (NAÏVE, CD24^+^IgD^+^CD27^-^) and memory (MEM, CD24^+^IgD^-^CD27^+^) B cells and plasmablasts/plasma cells (PB/PC, CD24^-^CD38^++^).

PBMCs from normal blood donors were incubated with the following anti-human Abs for 20 minutes at 4°C: V500 anti-CD19 (BD Biosciences), PerCPcy5.5 anti-CD38 (BioLegend), PE-cy7 anti-CD24 (BioLegend), FITC anti-IgD (ThermoFisher), and allophycocyanin anti-CD27 (BD Bioscience), and then sorted to isolate Transitional (TRANS; IgD^+^CD27^-^CD10^+^CD38^+^), NAIVE (CD27^-^IgD^+^), recirculating Marginal Zone (rcMZ; IgD^+^CD27^+^ (24), MEM (IgD^-^CD27^+^) and double negative (DN; IgD^-^CD27^-^) B cells.

Total CD19^+^IgD^+^CD27^-^ B cells were sorted from umbilical cord blood samples. In both cases, non-B cells were excluded with efluor-450 anti-CD3 and anti-CD16 mAbs, and dead cells barred with Sytox Blue (ThermoFisher) staining.

For all samples, B cells were sorted directly into 200µl PCR tubes containing 100µl Dynabeads Oligo(dT) (ThermoFisher) lysis buffer and stored at -80°C.

### Library preparation and sequencing

mRNA isolation from B-cell lysates was performed in 96-well plates using Dynabeads Oligo(dT) (ThermoFisher) according to the manufacture’s protocol. mRNA was used in its entirely for reverse transcription in 10 µl (50°C 1h, 72°C 10min) using SuperScript III Enzyme (ThermoFisher) in solid phase with Dynabeads Oligo(dT) as primer. After RNase H treatment, second-strand synthesis was performed (37°C 20 min, 98°C 30s, 62°C 2min and 72°C 10min) in solid phase in 10µl using Q5 Polymerase (NEB) and a mix of 13 primers covering all *IGHV* leader sequence segments reported in the IMGT database (25); primers contained a maximum of one mismatch, along with 13 to 16 random nt and partial Illumina adaptor sequences. Double-stranded cDNA was washed 3 times in 10mM tris-HCl to remove the remaining primers, and the entire sample was utilized as template for PCR amplification in 10 µl using Q5 Polymerase with universal FW primer and mix of reverse isotype specific primers (98°C 30s; 10 cycles at 98°C for 10s, 58°C for 15s, and 72°C for 1min; 72°C 10min). Two µl of the PCR product were used for a semi-nested PCR with inner RV primers for the constant region which also introduce partial Ilumina adaptors. This reaction was carried out in 20µl (98°C 30s; 15 cycles at 98°C for 10s, 58°C for 15s, and 72°C for 1min; 72°C 10min). The PCR product was purified with Ampure XP beads at a ratio of 1:1, and 1 - 10ng were used to add Illumina Indices with Nextera XT kit (Illumina). The MiSeq Illumina (v3 2 x 300 kit, Illumina MS-102-3003) was used to sequence the library. The library was loaded at 12pM with 10% PhiX [14].

### 9G4 Antibody labeling

The 9G4 rat anti-IGHV4-34 mAb (26) was labeled with Alexa Fluor ™ 488 according to the manufacturer’s recommendations (Alexa Fluor™ 488 Antibody Labeling Kit, ThermoFisher). Briefly, antibody solution was mix with1/10^th^ of 1M sodium bicarbonate and then incubated with Alexa Fluor™ 488 dye for 1h at room temperature. After the recommended period, the solution was placed in the provided purification column and labeled antibody was collected from the flow through.

### Analysis of IGHV4-34 IG heavy and light chain rearrangements in single B cells

To analyze the paired IG heavy and light chain IGHV-D-J and IGLVκJκ sequences of B cells expressing IGHV4-34, we used 10x methodology. After exposing normal Naïve, MZ, MEM, and DN B cell populations to the Alexa Fluor ™ 488-labeled 9G4 mAb, labeled cells were enriched by FACS (**Figure S6A**). For each sample, cells were washed and resuspended in 31.7 ul of PBS (0.04% BSA) immediately after sorting. Single-cell libraries were then generated using the Chromium Controller, Chromium Single Cell 5’ Library & Gel Bead Kit v2 and i7 Multiplex Kit (10x Genomics, Pleasanton, CA, USA), according to the manufacturer’s protocols. Target enrichment from cDNA was performed using the Chromium Single Cell V(D)J Enrichment Kit, Human B Cell (10x Genomics), followed by adaptor ligation. Enriched libraries were quantified on an Agilent Bioanalyzer High Sensitivity chip, and then sequenced on an Illumina Nextseq500 instrument (Illumina, San Diego, CA, USA) with the paired-end (2x150bp) mid output kit (300 cycles) according to manufacturer’s protocols.

### Bioinformatic analysis of immunoglobulin repertoire

For bulk VDJ-seq, processing of raw reads was performed using a custom workflow built with pRESTO (REpertoire Sequencing TOolkit) (27). The IGHV-D-J sequences obtained were submitted to IMGT/HighV-QUEST and analyzed using ChangeO and custom R scripts (23, 27).

Cellranger vdj pipeline was used to analyze sequencing data obtained from 10x Chromium V(D)J libraries.

### Attribution of IGHV-IGHD-IGHJ rearrangements to stereotyped CLL subsets

IGHV-IGHD-IGHJ gene rearrangements were analyzed for similarity to stereotyped CLL BCRs using our established bioinformatics method (10). In more specific, the subsequent clustering criteria were applied: (i) utilization of IGHV genes belonging to the same phylogenetic clan, (ii) ≥ 50% amino acid identity and ≥ 70% similarity within the VH CDR3, (iii) equal VH CDR3 length and, (iv) identical offset of the common amino acid motif.

Satellite CLL-SLS, i.e. sequences with strong immunogenetic similarities with major CLL subsets, were identified using a purpose-built bioinformatics algorithm, which is based on a set of previously described parameters (10) (1): utilization of phylogenetically associated IGHV genes (2), maximum VH CDR3 length difference of 2 amino acids, and (3), presence of the “subset-specific” VH CDR3 sequence motifs with an offset of ± 2 amino acids. This analysis was performed individually for each major subset.

### Statistical analyses

Statistical analyses and identification of outliers were performed in Graphpad Prism 9. Tests for statistical significance are described in figure legends for the relevant graphs.

## Results

### Identification of CLL stereotyped IGHV-D-J sequences in the B-cell repertoires of normal individuals

First, we asked if and to what extent B lymphocytes expressing BCRs closely resembling CLL-stereotyped BCRs exist in healthy people. To do so, we collected samples from CB of neonates (n=5), BM (n=11) of elderly people (≥ 70 years of age) who had undergone hip replacement surgery, and peripheral blood mononuclear cells (PBMC) of adult volunteers (35-60 years of age; n=16) (**Figures S1A, B**).

Total CD19^+^ B cells were FACS isolated from CB. Whereas from BM we isolated several B-cell subsets representative of the distinct B-cell developmental stages, using a combination of surface membrane markers (**Figure S1A**).

**Figure 1:**
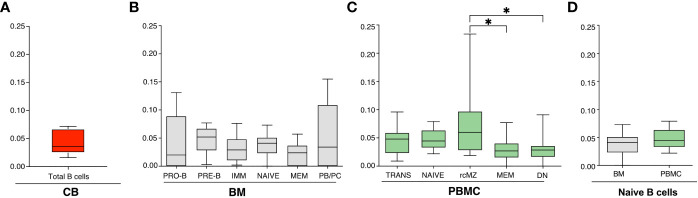
**(A-C)** Frequencies of CLL-SLS resembling standard stereotyped CLL BCRs identified in: **(A)** CB B cells (n=5); **(B)** BM B cell subsets (n=11); and **(C)** PBMC B cell populations (n=16). Statistical analyses were calculated with Kruskal-Wallis test. **(D)** Comparison of CLL-SLS in naïve B cells from BM and PBMC. Bars display minimum and maximum values. * p ≤ 0.05.

From each of the 3 different cell sources (CB, BM, PBMC), we were able to identify B cells bearing an IGHV-D-J rearrangement that corresponded to that of a known CLL stereotyped subset. For convenience, we refer to this type of rearrangement found in B cells from normal individuals as a “CLL stereotype-like sequence” (CLL-SLS). **Table S1** summarizes the number of unique CLL-SLS obtained for each cell population sorted from the various sites. We identified a total of 123, 513, and 999 CLL-SLS in CB, BM, and PBMC, respectively. The average frequencies were relatively comparable among the three (CB: 0.044%; BM: 0.037%; PBMC: 0.051%) (**Figures 1A**, **S1C**).

We then compared the distribution in BM of CLL-SLS at the various stages of B-cell development and among mature B-cell subsets. The highest frequency values were found among B-cell populations representative of the first stages of maturation: PRO-B, PRE-B, IMM, and NAIVE B cells (average frequencies: 0.046%, 0.048%, 0.031%, and 0.037%, respectively). The average frequency of CLL-SLS in MEM (0.023%) was lower, although these values did not reach statistical significance despite the number of total sequences queried being similar (**Table S1**). Interestingly, the PB/PC population harbored cells carrying CLL-SLS rearrangements at higher frequencies than immature/naïve stages (0.059%) (**Figures 1B**, **S1D**).

CLL-SLS were identified in each of the circulating B-cell subsets in PBMC: TRANS, NAIVE, rcMZ, MEM, and DN B cells (average frequencies: 0.047%, 0.047%, 0.076%, 0.032%, and 0.03%, respectively) (**Figures 1C**, **S1B**). CLL-SLSs were significantly higher in rcMZ compared to MEM and DN B-cell subsets (rcMZ vs MEM and DN, *P* ≤ 0.05) (**Figures 1C**, **S1E**). Lastly, we did not find a difference of CLL-SLS frequencies between NAÏVE from BM and NAÏVE from PBMC (**Figure 1D**).

In summary, normal B cells expressing standard CLL-SLS exist in the repertoires of healthy subjects and are present at similar frequencies from tissues that dramatically differs in age. Moreover, CLL-SLS are found at all stages of B-cell development, but, interestingly, at significantly higher frequencies in the PB/PC compartment in the BM and significantly higher frequencies in the rcMZ B-cell subset isolated from PBMCs.

Together, these findings suggest that CLL-SLS are not triaged during first stages of B cell development by clonal deletion. Since our data are generated from whole B-cell subpopulations, we cannot determine if receptor editing could have occurred in these dells, which also would allow them to transit through B-cell maturation. In addition, their presence in antigen-experienced B cell subsets, e.g., MZ and MEM, and among antibody-secreting B cells in the BM, could represent class switch recombination (CSR) and SHM along with positive selection by a particular antigen.

### Identification of satellite CLL-SLS in the B-cell repertoire of normal individuals

**Figure 2:**
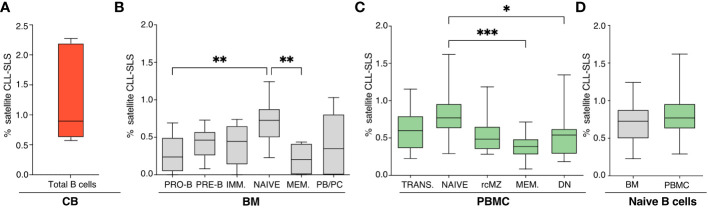
**(A-C)** Frequencies of CLL-SLS resembling satellite stereotyped CLL BCRs identified in: **(A)** CB B cells (n=5); **(B)** BM B cell subsets (n=11); and **(C)** PBMC B cell populations (n=16). Statistical analyses were calculated with Kruskal-Wallis test. **(D)** Comparison of CLL-SLS frequencies in naïve B cells from BM and PBMC. Bars display minimum and maximum values. * p ≤ 0.05, **p ≤ 0.01, ***p ≤ 0.001 .

Next, we extended our analysis to IGHV-D-J sequences that resemble those referred to as “satellites” of known CLL stereotyped subsets (10). Satellite stereotyped sequences resemble the VH CDR3 motif of CLL stereotyped subsets but differ in certain amino acid residues at specific positions in the IGHV-D-J rearrangement or vary in VH CDR3 length. Although satellites are only in a minor component of the total number of stereotyped subsets found in CLL patients (10), we identified a ≥ 10-fold enrichment of “satellite” CLL-SLS compared to standard CLL-SLS in every tissue (CB: 3,545 vs 123; BM: 6,145 vs 513; PBMC: 11,707 vs 999; **Tables S1, S2**). Notably, the highest frequency of satellite CLL-SLS was measured in CB, where they reached an average frequency of 1.31% of the total IG sequence (**Figures 2A**, **S2A**).

When examining satellite CLL-SLS at the different stages of B-cell development, we were able to detect satellite sequences in every B-cell subset isolated from the BM (PRO-B: 0.27%; PRE-B: 0.42%; IMM: 0.40%; NAÏVE: 0.70%; MEM: 0.20%; PB/PC: 0.39%). Differently from the standard we observed a statistically significant enrichment in satellite CLL-SLS in BM NAÏVE compared to BM PRO-B (*P* = 0.0089) and NAÏVE to PBMC MEM (*P* = 0.0016) (**Figures 2B**, **S2B**).

Similarly, in PBMC samples, satellite CLL-SLS were present at discrete frequencies inn all the BB cell subsets analyzed but we only observed a significant difference in satellite CLL-SLS frequencies when comparing the average of NAÏVE to MEM and DN cells (NAÏVE: 0.798 vs. MEM: 0.37%, *P* = 0.0007; NAÏVE vs DN 0.61% vs MEM: 0.37%, *P* = 0.0011; **Figures 2C**, **S2C**).

Like what we observed in the case of standard CLL-SLS, we did not detect any differences in the frequencies of satellite CLL-SLS when comparing NAÏVE from BM and PBMC samples (**Figure 2D**).

Thus, we identified satellite CLL-SLS in the B-cell repertoires of healthy donors. However, unlike CLL patients, where satellite subsets comprised only 3% of the total cohort versus 13.5% assigned to major subsets (10), satellite CLL-SLS in normal individuals were found at higher frequencies and numbers than standard CLL-SLS. This difference might be the result of their immunogenetic properties together with the selection forces shaping the normal B cell repertoire.

Finally, the highest frequency of satellite CLL-SLS was found in NAÏVE B cells coming from BM and PBMC, in contrast to what we observed in the context of standard CLL-SLS where the highest frequencies were found in PB/PC and rcMZ. Thus, similarly to what observed for standard CLL-SLS, the presence of satellite CLL-SLS distributed along all B cell differentiation axis further strengthens the idea that CLL-like BCRs are not subjected to elimination by central tolerance mechanisms and are present in the normal B-cell repertoire.

### Assignment of the CLL-SLS in normal, healthy people to specific, standard CLL stereotypes

**Figure 3:**
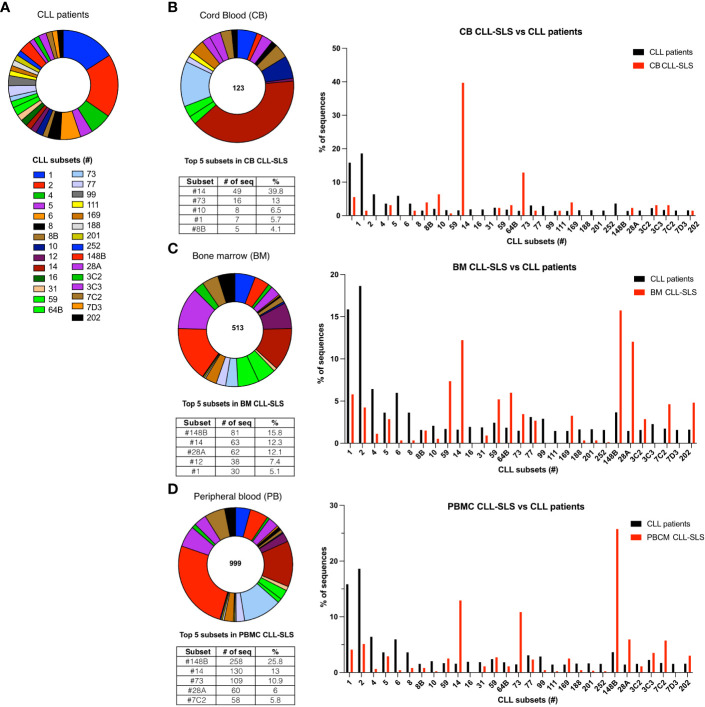
**(A)** Frequency distribution of the 29 different major standard stereotyped subsets among CLL patients. Each pie slice identifies a specific subset in a different color. **(B)** Left panel: Frequency distribution of the standard CLL-SLS in the CB. Each pie slice identifies a specific subset following color code in **(A)** Table below the chart indicates the top 5 most frequently found standard CLL-SLS. Right panel: Comparison of the frequencies of major CLL stereotyped subsets in patients and in CLL-SLS from CB. **(C)** Left panel: Frequency distribution of the standard CLL-SLS in the BM. Right panel: Comparison between the frequencies of major CLL stereotyped subsets in patients and in CLL-SLS from BM. **(D)** Left panel: Frequency distribution of the standard CLL-SLS in the PBMC. Right panel: Comparison of the frequencies of major CLL stereotyped subsets in patients and in CLL-SLS from PBMC.

The second tier of analysis was directed at understanding to which specific standard stereotyped CLL subset the CLL-SLS belong, and at determining if the distribution of the CLL-SLS differs from the standard stereotyped sequences observed in cohorts of CLL patient (**Figures 3A–D**). When comparing the distribution of standard CLL-SLS resembling the 29 most prominent subsets identified in patients with CLL across the 3 different cell sources (**Figures 3B–D**), we found at least one sequence belonging to each of the major CLL stereotyped subset from each site apart from subsets #16 and #7D3 (**Table S3**). When examining CB B cells, 39.8% of the CLL-SLSs were members of subset #14 and 13.01% of subset #73 (**Figure 3B**). Together these two subsets made up ~50% of all the CLL-SLS in the CB. Interestingly, these subsets were not the most prevalent ones among the major CLL subsets (**Figure 3A**). Finding these at increased frequencies in the CB, where most B cells have not encountered foreign antigen and have not undergone somatic hypermutation (SHM), was also surprising because, among CLL patients, these two subsets display mutated IGHVs. However, for subsets # 73 and 14, most of the CLL-SLS sequences were IGHV-unmutated (75% and 84%, respectively).

In the BM, the most recurrent subsets were # 148B, #14, #28A, #12 and #1 (15.8%, 12.3%, 12.1%, 7.4% and 5.1 respectively; **Figure 3C**). Like the CB, these abundant subsets were not the most frequently found in patients (**Figure 3A**). CLL B cells belonging to subsets #1 and #28A are part of the IGHV-unmutated (U-CLL) group and are encoded by IGHV1-69 (subset #1) and IGHV1-2 (subset #28A), respectively. Conversely, CLLs falling into subsets #148B and #14 express IGHV2-5 and IGHV4-4, respectively, and belong to the IGHV-mutated (M-CLL) group. For those CLL-SLS identified among PBMCs, again subsets # 148b and #14 were highly represented (25.8% and 13%), followed by subsets # 73, #28A and #7C2 (10.9%, 6.0, and 5.8% of the total CLL-SLS) (**Figure 3D**).

Finally, when comparing among the three tissues, we found that BM and PBMC display a very similar distribution of CLL-SLS with a statistically significant difference in the frequencies only for subset #148b (**Figure S3A**).

In conclusion, the distribution of certain standard CLL-SLS found in normal individuals can be dramatically different from the distribution found in CLL patients. However, since little is known about the immunogenetic properties of most of the subsets found frequently in the normal repertoire, it is difficult to speculate about the underlying biological process or force that preferentially selected for a B cell expressing a particular stereotyped sequence. In addition, we did not observe major differences in the distribution of CLL-SLS subsets when comparing BM and PMBC samples. Nevertheless, we noticed a discrete difference when comparing these to CB sample, where 50% of CLL-SLS are attributed to two subsets. Finding such high-level restriction in the CB is not necessarily paradoxical, given the absence of terminal deoxynucleotidyl transferase expression with the consequent lack of non-templated additions during the neonatal period, which often significantly limits diversity in the VH CDR3.

### Assignment of CLL-SLS found in normal individuals to specific satellite CLL stereotypes

**Figure 4:**
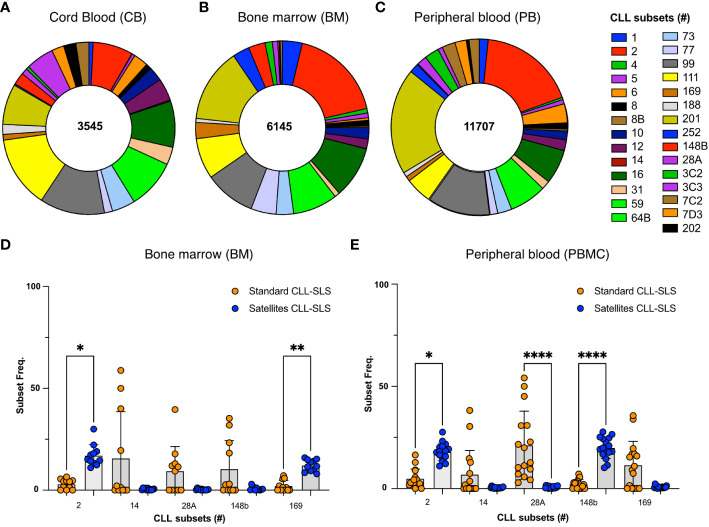
**(A-C)** Pie chart displays the frequency distributions of satellite CLL-SLS in **(A)** CB; **(B)** BM. Each pie slice identifies a specific satellite stereotyped subset following color code in the legend. **(D, E)** Plot summarizes the changes in frequencies of the depicted subsets between standard and satellite CLL-SLS in **(D)** BM and **(E)** PBMC. Statistical analyses were performed with Kruskal-Wallis test. * p ≤ 0.05, **p ≤ 0.01, ****p ≤ 0.0001, # = subset number.

We next analyzed the distribution of satellite CLL-SLS as above. This identified several differences from that found for the standard CLL-SLS. For example, in CB, 50% of CLL-SLS were satellites of subsets # 77, #73, #31, #12, #2, and #169 (**Figure 4A** and **Table S3**). Within the BM, we also found that the most recurrent subsets were # 2, #169, #12, and #73 (17.5%, 12.9%, 8.8%, 9.1%, respectively; **Figure 4B**). Similarly, for the peripheral blood samples, subsets # 2 and #169 were among the most frequent (19.1% and 21.1%, respectively), making up to 40% of the total sequences (**Figures 4C**, **S4A**). It is noteworthy that the satellite sequence for subset #169 was found among the top-ranking group for each of the three sites of collection.

Furthermore, when we compared the frequencies of standard and satellites subsets, we observed two different scenarios. For some specific subsets we found a significant increase in satellite CLL-SLS compared to the standard ones. For example, #169 and its companion #2 comprise 30 to 40% of all the sequences in PBCM and BM whereas only a minor fraction of standard CLL-SLS were attributed to subset #2 and #169 in both tissues examined (BM: #2, 2.9% and #169, 2.5%; PBMC: #2, 4.8% and #169, 2.3%) (**Figures 4D, E**). However, we also found an opposite behavior for some of most frequent standard subsets that instead were underrepresented in the satellites, e.g., # 148b, 28A, and 14 (**Figures 4D, E**).

Overall, the satellite CLL-SLS subsets enriched in specific B-cell subpopulations can differ significantly from the standard CLL-SLS, especially in PBMC and BM. Among individual satellite subsets, # 2 and #169 seem to be over-represented in both PBCM and BM, and they both significantly increase in frequencies when compared to their standard subset counterparts. Alternatively, some of the most frequent subsets in the standard CLL-SLS analysis were underrepresented in the satellite analysis. This last observation is in accord to what is observed in CLL patients, where satellite represent only a minor fraction of the total subset sequences. Instead, the significant increase in satellite CLL-SLS attributed to #2 and #169 suggest a selection pressure of these type of sequences compared to the standard counterpart that only occurs in the normal repertoire.

### Distribution of CLL-SLS corresponding to IGHV-unmutated and IGHV-mutated CLL subsets among B-cell subpopulations at different stages of B-cell development

**Figure 5:**
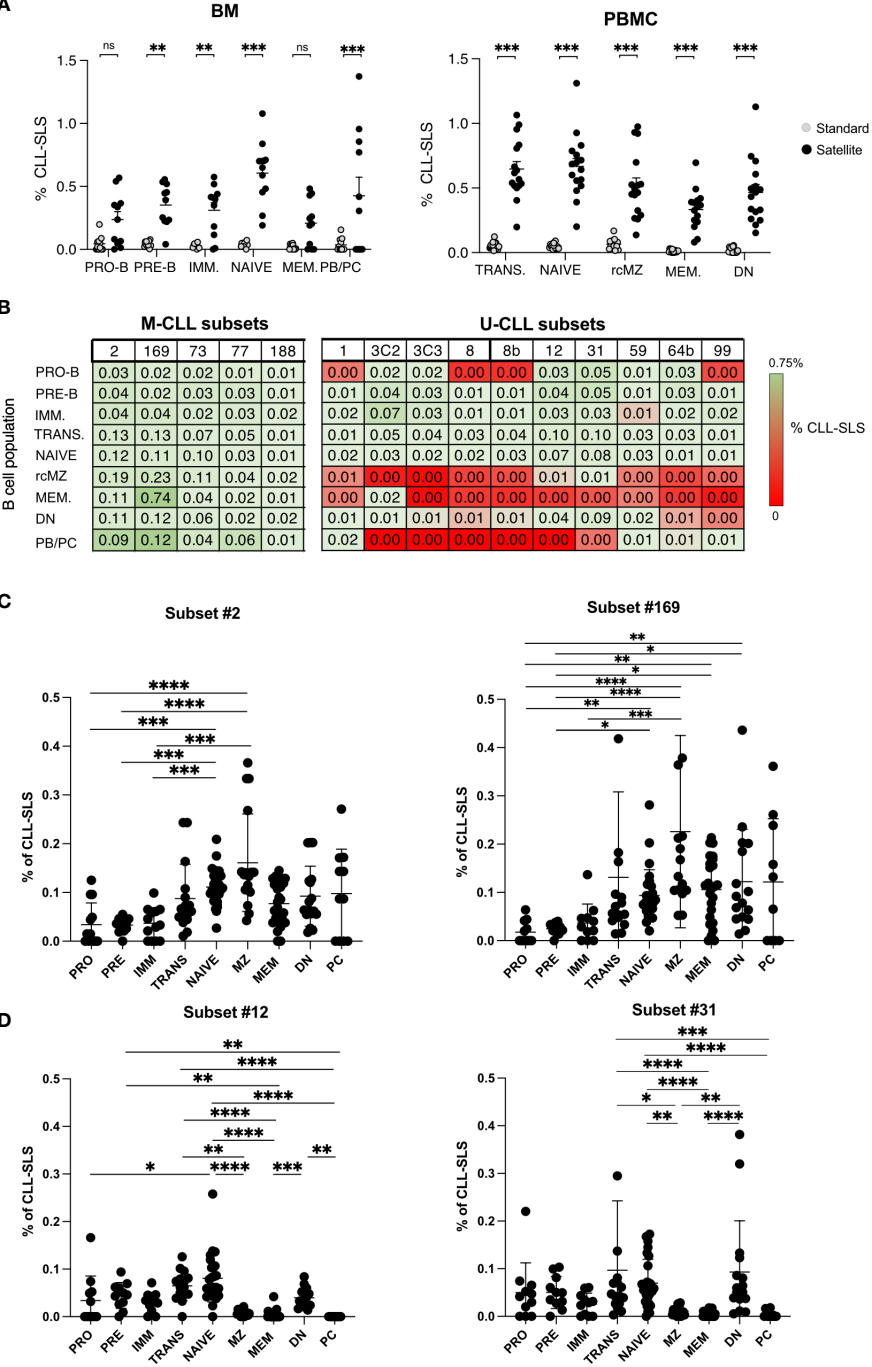
**(A)** Left Panel: Summary plot comparing frequencies of standard and satellites CLL-SLS in different B cell subsets in BM. Right Panel: Summary plot comparing frequencies of standard and satellites CLL-SLS in different B cell subsets in PBMC. **(B)** Upper panel: Table summarizes average frequency of the most frequent satellite CLL-SLS divided by subsets through different stages of B cell development. Lower panel: Table summarizes average frequency of the less frequent satellite CLL-SLS divided by subsets through different stages of B cell development. **(C)** Summary plot of satellite CLL-SLS frequencies attributed to subset #2 and #169 through different stage of B cell development (Mean with SEM). Statistical analysis and multiple comparisons were performed with Kruskal-Wallis test. **(D)** Summary plot of satellite CLL-SLS frequencies attributed to subset #12 and #31 through different stage of B cell development (Mean with SEM). Statistical analysis and multiple comparisons were performed with Kruskal-Wallis test. * p ≤ 0.05, **p ≤ 0.01,***p ≤ 0.001, ****p ≤ 0.0001. ns, not significant.

Like CLL clones (4), stereotyped subsets can be segregated based on IGHV-mutation status (10,28). Therefore, we next determined if the U-CLL CLL-SLS were enriched in the earlier stages of B-cell development and if the M-CLL CLL-SLS were enriched in the later stages of B-cell maturation (**Figures 5B, C**). Since the incidence of standard CLL-SLS differed significantly from satellite CLL-SLS, for the following analysis, we only analyzed the former (**Figure 5A**).

Since we did not find proof for negative selection of any individual CLL-SLS at the early maturation stages in the BM and at the transitional stage in the blood (**Figure 5B**), we checked for such evidence at later stages of maturation, by examining specific patterns of distribution of individual CLL-SLS. This analysis revealed that CLL-SLS belonging to the IGHV-mutated subsets were found at different frequencies than the IGHV-unmutated among the various antigen-experienced B cell subsets (**Figures 5B, C**).

Using subsets #2 and #169 as examples of IGHV-mutated standard subsets, for subset #2, there was a statistically significant enrichment in NAIVE and rcMZ compared to B cells at the earlier stages of development; a significant level of different was not found with B cells at the later stages of maturation, although there was a trend in this regard. The principal was the same for subset #169, except there were also statistically significant differences for the MEM and DN. There was a similar trend for enrichment in PC/PB, but this did not reach statistical significance. Since subsets # 2 and #169 are members of the IGHV-mutated subtype, this pattern of distribution in the mature stages of B-cell development seems consistent. Similar trends were found for M-CLL subsets # 73, #77, and #188 (**Figure S5A**).

In contrast, those CLL-SLS expressing unmutated IGHVs were detected in B-cell subpopulations at the early stages of development and in TRANS and NAIVE; they were virtually absent from the IGHV-mutated MEM and rcMZ populations. Examples are CLL-SLS belonging to U-CLL subsets # 12 and 31 (among the most frequent ones, **Figure 5D**); a similar trend was observed for subsets # 8, 8B, and 59 (**Figure S5B**). Surprisingly, however, CLL-SLS attributed to U-CLL subsets were significantly enriched in DN B cells, an Ag-experienced, usually IGHV-mutated B cell subpopulation that plays a role in auto-reactive conditions and infectious disease (29). The reason for the abundance in this IGHV-mutated B-cell subpopulation is not obvious.

In summary, when examining individual CLL-SLS subsets, CLL-SLS bearing IGHV-mutated IGs are most frequent in naïve and Ag-experienced B cells, whereas CLL-SLS attributed to IGHV-unmutated subsets in general are restricted to the early stages of B-cell maturation and to naive B cells, with DN B cells being the exception.

### Pattern of SHM found among CLL-SLS from normal, healthy people

**Figure 6:**
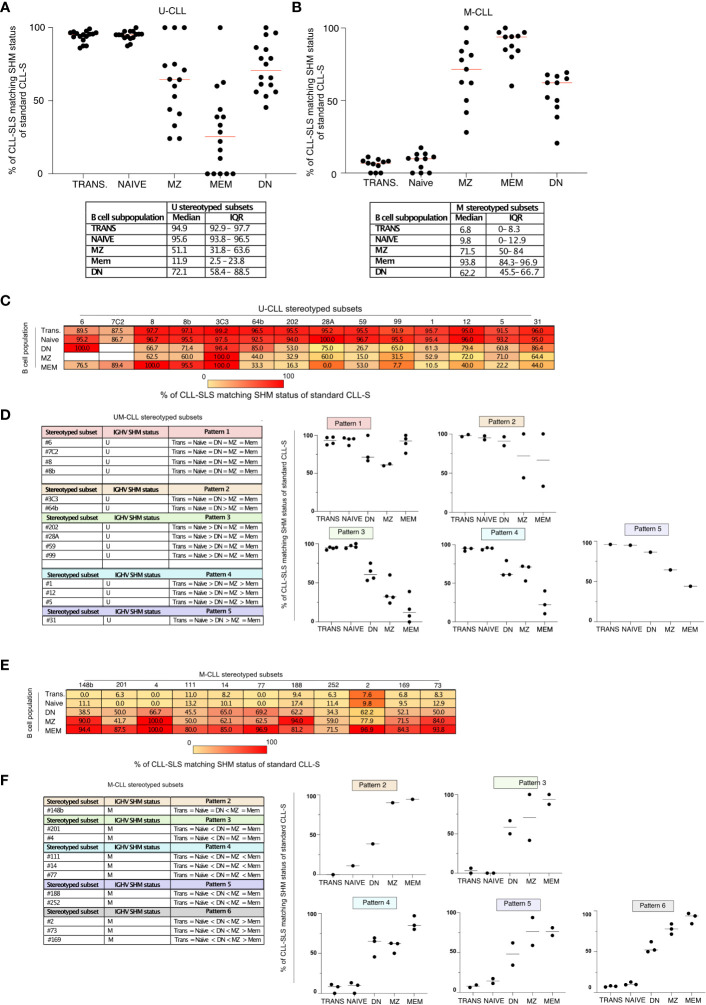
**(A)** Summary plot of the frequencies of IGHV-unmutated CLL-SLS with matching U-CLL stereotyped subsets (median). **(B)** Summary plot of the frequencies of IGHV-mutated CLL-SLS with matching M-CLL stereotyped subsets (median). **(C)** Table with median frequencies of IGHV-unmutated CLL-SLS with matching U-CLL stereotyped subsets divided by subsets. **(D)** Left panel: CLL-SLS attributed to U-CLL subsets were grouped in pattern based on the matching frequencies. Right panel: Plots showing the median frequency of CLL-SLS matching U-CLL status. Each plot represents one of the patterns indicated in the left panel. **(E)** Table with median frequencies of IGHV-mutated CLL-SLS with matching M-CLL stereotyped subsets divided by subsets. **(F)** Left panel: CLL-SLS attributed to M-CLL subsets were grouped in pattern based on the matching frequencies. Right panel: Plots showing the median frequency of CLL-SLS matching M-CLL status. Each plot represents one of the patterns indicated in the left panel.

Next, we examined if the CLL-SLS enriched at different B-cell maturation stages in PBMCs exhibited the same IGHV-mutation status as that found in standard CLL stereotyped subsets (**Figures 6A, B**).

First, we analyzed CLL-SLS attributed to U-CLL subsets, observing that ~94% of CLL-SLS found in TRANS and NAIVE B cells matched the IGHV-mutation status of the stereotyped subsets in CLL patients (**Figure 6A**). This situation changed when examining the more mature B-cell subsets in PBMC. In the case of MZ and DN, there was a fall in the frequencies of IGHV-unmutated CLL-SLS matching the IGHV-mutation status of the patient-defined CLL subsets (61% and 72%, **Figure 6A**). This drop was most evident for CLL-SLS in MEM B cells, where 72% displayed SHM and only 28% matched the original SHM status (**Figure 6A**).

When examining CLL-SLS belonging to M-CLL subsets (**Figure 6B**), we found the opposite: only a minor fraction present in the NAIVE and TRANS B-cell compartments displays SHMs and hence matches the IGHV-mutation status in CLL (8% and 5.7%, respectively). The majority in MEM (88.4%), MZ (68%), and DN (54.5%) were mutated and thus in agreement with CLL SHM status.

Thus, CLL-SLS that are IGHV-unmutated in patients are found more often in the normal repertoire among B cells at the earlier stages of B-cell maturation, which have usually not interacted with foreign antigens and therefore have not undergone SHM and developed IGV mutations. In contrast, CLL-SLS that are IGHV-mutated in patients are found more often in the normal repertoire among B-cell subpopulations at the later stages of B-cell maturation when SHM is common. So, in general, these results are consistent with U-CLL clones originating from and TRANS, NAÏVE, and MZ B-cell populations, and M-CLL clones coming from more antigen-experienced subsets such as MZ, MEM, and DN. However, the fact that some U-CLL-associated CLL-SLS can bear somatic IGHV mutations and can be enriched in antigen-experienced B cells suggests a positive selective driving away from the corresponding CLL-associated IGHV-mutation phenotype for these CLL-SLS.

We next expanded this type of analysis to individual CLL-SLS from the major CLL stereotyped subsets, examining the distribution patterns of U-CLL-like and M-CLL-like SLS sequences among the different B cell subpopulations (**Figures 6C–F**). This revealed 7 distinct patterns (**Figures 6C–F**). In the case of U-CLL subsets exhibiting pattern 1, the vast majority of IGHV-unmutated CLL-SLS belonging to subsets # 6, #7C2, #8, and #8B match the IGHV-mutation status of the CLL patients, independent of the B-cell subset in which they were found (**Figures 6C, D**). Regarding the other patterns, most were still characterized by high frequencies of CLL-SLS matching the SHM pattern of CLL patients; these were mainly restricted to the NAIVE and TRANS B cell stages. However, there were obvious differences in the other B-cell subsets. For example, in the case of subsets belonging to pattern 3 (subsets #202, #28A, #59, and #99), only a median of 65% DN, 32.9% MZ, and 7.7% MEM CLL-SLS matched the original CLL IGHV mutation status. Similarly, by looking at CLL-SLS attributed to M-CLL subsets, we observed that the most frequent ones found in our analysis, such as # 2, #73, and #169, group together in pattern 6 (**Figures 6E, F**). In this case, MEM display the highest median frequencies of CLL-SLS matching the original CLL IGHV mutation, whereas there was a progressive decline in both MZ and DN populations. In other cases, such as those subsets belonging to pattern 5, both MZ and MEM were similarly enriched in CLL-SLS matching the IGHV mutation status of the original CLL subsets (**Figures 6E, F**).

Thus, when examining the SHM status of individual CLL-SLS, different patterns can be identified. These patterns suggest that specific CLL stereotyped subsets might originate from particular subpopulations in the normal B-cell repertoire.

### More precise documentation and assignment of CLL-SLS to normal B-cell subsets based on expression of IGKV and IGLV genes

The preceding data indicate that CLL-SLS are present in normal, healthy people, and that the frequencies at which these exist in the normal B-cell repertoire do not appear to decrease when progressing from developing to mature B cells. Since CLL B cells appear to derive from autoreactive precursors (13) and CLL IGs are often autoreactive (13–17), finding CLL-SLS in healthy individuals at all stages of maturation is not consistent with the elimination of autoreactive BCRs/IGs from the normal B-lymphocyte repertoire (18). Alternatively, the IGK/LV-J rearrangements paired with the CLL-SLS IGHV-D-J could differ from those in CLL cells, thereby neutralizing or preventing autoreactivity. Since several of the major stereotyped subsets display IGK/LV gene restrictions, we isolated cells based on membrane L chain expression (κ or λ) and defined the frequencies at which certain specific CLL-SLS IGHV-D-J rearrangements were found in the κ and λ chain populations.

**Figure 7:**
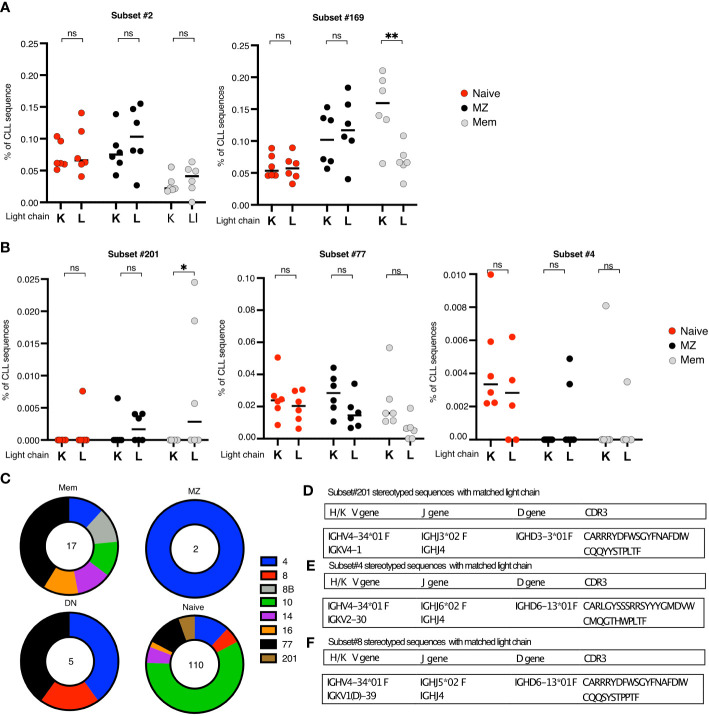
**(A, B)** Summary plots comparing the mean frequencies of CLL-SLS in the IG κ and IG λ fractions of the indicated B cell populations. Each plot refers to a particular CLL subsets. Statistical analysis and multiple comparisons were performed with Anova, Holm-Sidak test. **(C)** Pie charts of the frequency distribution of the CLL-SLS found after single cell RNA sequencing of the indicated B cell population. Each pie slice identifies a specific satellite subset following the color code in the legend. **(D-F)** Table of features of IGHV and matching IGK/LV attributed to **(D)**. subset #201; subset #4; Subset #8. *p ≤ 0.05, **p ≤ 0.01, ns, not significant.

As representatives of those subsets that display IGK/LV L chain restriction, we examined subset #2 and its companion subset #169, both of which always express lambda light chains encoded by IGLV3-21. Notably, when sorting NAIVE, MZ, and MEM cells from a normal, healthy person based on L chain isotype, we observed a similar distribution of CLL-SLS subsets # 2 and 169 within the κ and λ chain expressing B-cell populations. Thus, for these subsets there was a lack of skewing toward λ light chain use (**Figure 7A**).

We then looked at CLL-SLS subsets characterized by the expression of IGHV4-34, such as subsets #201, #77, and #4 (**Figure 7B**). For subset #201, which always uses IGLVλ1-44, we found CLL-SLS only in the λ-expressing fraction of MEM B cells. Whereas for subsets # 77 and 4, whose IGHVs always pair with IGLV10-54 or IGKV2-30, respectively, we did not observe a particular bias toward the usage of a specific light chain type (**Figure 7A**). Thus, we found the CLL-SLS in the appropriate light chain population depending on the specific subset. However, by taking this approach, we could not determine the specific IG light chain expressed by CLL-SLS.

To overcome this challenge, we performed single cell IGHV-D-J sequencing of B cells in PBMCs that express IGHV4-34 by sorting using the 9G4 mAb which reacts specifically with IGs bearing this gene (30). This strategy allowed us to enrich for subsets that use IGHV4-34 and, at same time, to identify the matching L chain and its DNA sequence. After sorting 20,000 NAIVE, 4,274 MZ, 9,547 MEM, and 340 DN B cells (**Figure S6A**), we identified IGHV-D-J sequences from 5,406 NAIVE, 1,205 MZ, 2,386 MEM, and 122 DN cells. A large majority of clonotypes identified expressed IGHV4-34, as depicted by V to J heatmap (**Figure S6B**). In this way, we identified CLL-SLS representative of several stereotyped subsets associated with IGHV4-34 (**Figure 7C**) in 110 Naïve, 2 rcMZ, 7 MEM, and 5 DN cells.

When looking specifically at CLL-SLS sequences resembling subset # 201, we identified 6 such sequences, all in naïve B cells. However, none were paired with a λ light chain (**Figure 7D**). However, although subset #201 CLL clones show a strong restriction for λ light chain use, there are a few identified instances where subset #201 stereotyped sequences were paired with the κ light chain gene IGKV4-1. Notably, one of our CLL-SLS sequences attributed to subset #201 had an IGKV4-1 gene partner. Moreover, the VK CDR3 sequence of that cell was remarkably like the CLL stereotype and the CLL-SLS (**Figure 7D**). Thus, this apparently normal B cell could be a precursor to a subset #201 CLL clone.

Similarly, when examining CLL-SLS resembling subset #4, we found an IGHV-D-J gene rearrangement paired with IGKV 2-30, the gene most often co-expressed in this leukemic subset (**Figures 7E, F**). However, this CLL-SLS did not bear IGHV mutations, which all subset #4 rearrangements have. Nor did it carry a characteristic amino acid at a specific position in the IGKV-J rearrangement corresponding to the standard stereotyped CLL BCRs, i.e., an aspartic acid at position 66 in the VK FR3 that is introduced by SHM in CLL cells. Thus, this apparent precursor of standard CLL stereotyped subsets # 4 does exhibit the complete subset #4 CLL sequence.

## Discussion

Using our efficient IGHV-D-J sequencing approach that provides considerable depth of analysis (23), we demonstrated that CLL-SLS are present in B lymphocytes from normal individuals isolated from three sources that differ in B-cell composition and age. In line with a recent report finding such rearrangements in fetal liver-derived B cells (31), our studies indicate that CLL-SLS are present at the first stages of developmental time, in our instance, human cord blood. Since the median age of diagnosis of CLL is ~70, it might be expected that samples from aged individuals would contain higher frequencies of CLL-SLS. Notably, however, despite the age differences in the sites we sampled, the frequencies of CLL-SLS in the CB, PBMC and BM were similar, suggesting that CLL-SLS accumulation does not change with aging.

In addition, when focusing on the adult PBMC and BM repertoires, we did not find a fall in the frequency of normal B cells bearing CLL-SLS suggesting that censoring by central tolerance mechanisms had not occurred in the BM This was surprising since CLL IGHV-D-J rearrangements, including stereotyped rearrangements, generally derive from autoreactive B cells that normally would be eliminated (32,33). This lack of censoring suggests that CLL-SLS do not recognize self-antigen with sufficient affinity to activate clonal deletion mechanisms. This conclusion, however, might be premature since, in the main, we analyzed solely IGHV-D-J rearrangements and not their accompanying IGKV-J and IGLV-J rearrangements, both of which are often needed for autoantigen binding. Thus, receptor editing of IGK/LV genes (18), another mechanism to maintain tolerance, could have taken place in B cell carrying CLL-like BCRs and could explain why CLL-SLS are relatively overabundant in normal people. Consistent with this possibility, certain major CLL stereotyped subsets use specific IGK/LV genes (34). So, this L chain feature might reflect the actions of an operative, normal tolerance mechanism and represent a level of negative selection for B cells bearing potentially harmful autoreactive BCRs in people without CLL. Finally, CLL-SLS might evade negative selection in the BM if the B cell carrying those rearrangement go through receptor revision of the H chain (35, 36).

We did find a fall in the frequencies of CLL-SLS among B-cell subsets in PBMC. Specifically, the highest level of such sequences was identified in NAIVE, TRANS, and rcMZ B cells with a decrease in the MEM and DN compartments. These observations suggest that CLL-SLS were not purged at the TRANS level, another point in early development where autoreactive B cells can be triaged from the repertoire (37, 38). Consequently, normal B cells bearing BCRs with acceptable, not intolerably high reactivity with autoantigens, could expand, possibly by tonic BCR signaling, and move into the mature B-cell pool (37–39). Hence, the lack of apparent negative selection at this stage would again be consistent with the CLL-SLS bearing lower affinity BCRs to autoantigens than registered by the immune system as dangerous.

However, the decrease in MEM and DN B cells suggests that the next set of immune tolerance mechanisms that prevent entry of unwanted, high affinity specificities B cells with into the more mature stages is effective in normal people. Hence, at least some CLL-SLS are prevented from engaging in germinal center-like responses. This could especially be the case for those CLL subsets using IGHV4-34, e.g., #4 and #201, since IGHV4-34-bearing normal B lymphocytes are usually excluded from GC reactions and prohibited to differentiate to antibody-secreting cells due their inherent autoreactive profile (40, 41). However, this does not totally exclude the possibility that processes such as SHM and CSR, occurring during GC reactions could redeem those potentially self-reactive CLL-SLS and allow them to mature to MEM and DN B cells. Lastly, CLL-SLS B cells that make it into the MEM and DN pool could become anergic, and therefore not increase numerically.

Finding cells with CLL-SLS BCRs in the MZ B subpopulation at frequencies higher than naïve could appear contrary to this principle. However, this might be explained by the features of the MZ B-cell subset. Indeed, even though IgM-expressing MZ B cells can be antigen-experienced, they can be generated through GC-independent process and they are poorly recruited to GC reaction and can be generated through a GC-independent process (42). Additionally, TRANS B cells can bypass the NAÏVE stage and differentiate in the MZ (43). Thus, high frequencies of CLL-SLS in the MZ B-cell compartment might be the combined result of a distinctive B-cell developmental route and of reduced involvement in GC reactions. This result differs from that reported recently, where CLL-SLS were present at significantly lower percentages in rcMZ B cells (21). This inconsistency might be due to different criteria and bioinformatics tools used to identify CLL-SLS.

In addition to those CLL-SLS attributed to standard stereotyped CLL subsets, we identified an unexpectedly high number of satellite CLL-SLS. Indeed, there was a ≥ 10-fold enrichment compared to standard CLL-SLS in every tissue. Interestingly, this is the opposite of what is seen in CLL patients, where satellites are only a minor component of the stereotyped subsets identified. A plausible explanation for the higher frequency of satellite CLL-SLS compared to the standard CLL-SLS is the more relaxed criteria used to identify satellite sequences. However, if that was the only factor involved, then the same observations would be also made in CLL. Hence, the frequency of standard and satellite CLL-SLS in our cohort could be the result of their immunogenetic properties together with selection forces shaping the normal B-cell repertoire.

Like standard CLL-SLS, satellite CLL-SLS were found at different frequencies throughout all stages of B-cell development with a significant enrichment in Naïve B cells in the BM and PBMC. This observation also suggests that satellite CLL-SLS are not subjected to negative selection during first steps of B-cell maturation. On the other end, a decrease in both MEM and DN B cells, is consistent with antigenic selection representing a barrier for B cells carrying CLL-like BCRs. Finally, this decrease might represent dilution of B cells bearing CLL-SLS in favor of positively selected non-CLL-SLS normal B cells by foreign antigen.

Regarding standard CLL-SLS, it was notable that those subsets found most recurrent in the CB, BM, and PB (#14, #73, #148b, #28A) were not those that are the most prominent in patients with CLL (#1, #2, #4, #6, and #8). Since there is not sufficient information available about the standard subsets found enriched in the normal repertoire, we can only propose that this distinction reflects a lesser necessity to remove or edit the former rearrangements and/or a greater need to remove the latter.

In this regard, it is noteworthy that the vast majority of CLL-SLS found in the CB are attributed to only two subsets (#14 and #73), both of which are in IGHV-mutated in CLL. However, most of the CLL-SLS sequences attributed to these two subsets are IGHV-unmutated in the CB.

Moreover, an uneven distribution of stereotyped subsets in the CB could reflect restrictions which, in many cases, are defined by unique combinations of IGHV, IGHD and IGHJ genes (‘germline motifs’) with less significant contribution by the IGHV-IGHD and IGHD-IGHJ gene junctions. Finding such high-level restriction in the CB is not necessarily paradoxical, given the absence of terminal deoxynucleotidyl transferase expression with the consequent lack of non-templated additions during the neonatal period, which can leads to severe limitations of diversity in the VH CDR3 (44, 45).

Of interest, when looking at satellite CLL-SLS, the findings were different. Strikingly, in both BM and PBMC, the most recurrent subsets identified were satellites of subsets #2 and #169, which fit into the set selected against in standard stereotyped instance, suggesting that negative selection for such satellite sequences had not occurred.

When assigning CLL-SLS to distinct normal B-cell subpopulations differing in foreign antigen experience based on IGHV mutations, we found some CLL-SLS predominate in subpopulations matching or not their IGHV-mutation status. For example, as expected, CLL-SLS attributed to the U-CLL type were mainly found in TRANS and NAIVE B-cell subpopulations (# 12, #31, #8, #8B and #59), whereas, unexpectedly, some CLL-SLS that do not carry mutations in CLL patients (#59 and #99) were highest in IGHV-mutated MEM cells. Likewise, CLL-SLS of the M-CLL type predominated in rcMZ, MEM, and DN (e.g., #201 and #4). Nevertheless, the majority of subset #2 and #169 CLL-SLS were IGHV-mutated and found in the MEM, and ~50% of the subset #2 and #169 CLL-SLS in the DN compartment exhibited somatically mutated BCRs. Thus, those B cells bearing BCRs that are discordant in IGHV-mutation status between the CLL setting and the normal setting would not be identified as the normal counterpart of leukemic clones. We can only speculate whether B cells carrying CLL-SLS that were found in M-CLL subsets but had discordant SHM status and were found in the NAÏVE compartment might represent a candidate precursor of CLL. Indeed, those cells have the potential to differentiate and accumulate SHMs and become identical to the original CLL counterpart.

As mentioned, several standard CLL-SLS can exhibit striking light chain gene sequence restriction. We took advantage of this issue by sorting B-cell subpopulations based on surface expression of κ or λ L chains and then asking if certain CLL-SLS, defined by the presence of either IGKV or IGLV genes in CLL, were enriched in normal B cells expressing that L chain isotype. Notably, for subsets # 2 and 169, we did not find such a restriction in L chain use as CLL-SLS attributed to these subsets were present in the λ-expressing and the κ-expressing fractions of the different B-cell populations sorted. Different, however, was the case for BCRs belonging to CLL subset #201, for which we found subset #201-like CLL-SLS only in the λ-expressing fraction of normal IgM memory B cells, consistent with the findings in patients with CLL. Thus, some CLL-SLS in normal B-cell populations express both the particular H and L chain subtypes reminiscent of a CLL cell and others do not. Subsets # 2 and 169, are examples of the latter and again suggest that these would not lead to CLL.

To formally address the possibility that a single normal B cell could express a CLL-SLS BCR IG carrying a single IGHV-D-J and IGKLV-J as found in patients with CLL, we performed single cell V(D)J sequencing analysis of B-cell populations sorted for surface membrane expression of IGHV4-34 using the 9G4 mAb. When checking IGHV4-34^+^ sequences bearing the subset #201 IGHV-D-J rearrangement, we did not find any of these paired with the expected λ gene. However, we did identify a subset #201 CLL-SLS along with a companion IGKV2-30 gene that is used in some CLL subset # 201 clones. However, this pair was only found in the naïve B-cell population, not in the more mature subsets that bear IGHV mutations as subset #201 usually does. Thus, finding a H-L BCR pair resembling the standard stereotype CLL BCR in the NAIVE but not in an antigen-experienced B-cell subset is consistent with effective peripheral tolerance censoring in normal individuals (40, 41).

Likewise, we identified other cells bearing the subset #4 CLL-SLS that were paired with the specific κ L chain gene rearrangement corresponding to that found in CLL. Interestingly, however, the expressed IGHV of this cell was not somatically mutated and did not carry a characteristic amino acid present at a specific position in the light chain variable region that is found in that standard stereotyped CLL BCR. Thus, in this instance, either there was a negative selection triaging against such specificities entering the mature B-cell repertoire or the antigenic drive needed to initiate these mutations did not occur in the normal setting.

These single cells analyses, thus, provided two examples of B lymphocytes within the B-cell repertoire of apparently normal people that differed in the potential to be a CLL precursor. The first example (CLL-SLS #201) was not consistent with this, suggesting either that the potential precursor was blocked from attaining or was negatively selected after attaining the canonical subset #4 CLL sequence. The second (CLL-SLS #4) is consistent with this finding and suggests that censoring of a CLL precursor does not necessarily occur. Single cell sequencing at depths greater than those we achieved will be necessary to determine which of these possibilities is correct.

Finally, finding certain, specific CLL-SLS in discrete normal B-cell populations raises the possibility that the final transformation event for that stereotyped subset occurred in that population or at that anatomic site. Thus, one could speculate that certain normal B-cell populations represent reservoirs in which specific stereotyped CLL clones are transformed. In this regard, SHM could act to control or promote CLL-SLS expression and transformation in the various mature B-cell repertoires. However, the possibility that transformation happens earlier but the transformed cells retain the ability to respond to specific types of antigens and to follow distinct maturation pathways, which lead to over or under abundance in distinct B-cell populations, cannot be excluded.

Collectively, our findings are consistent with CLL stereotypes not being sufficiently autoreactive to be censored by central and early (TRANS level) tolerance mechanisms, and therefore being permitted to enter the NAIVE subpopulation. Nevertheless, after arriving in the NAIVE repertoire, peripheral tolerance mechanisms for some CLL-SLS appear to restrict the number of cells entering the more mature B-cell repertoires in normal individuals, except possibly for the MZ. In patients with CLL, however, the later tolerance checkpoints might be faulty, allowing these CLL-SLS to be enriched in antigen experienced and memory B cells. In addition, some of those B cells with BCRs resembling those in CLL that do differentiate to MEM, DN, and PB/PC do not necessarily exhibit a H-L pairing, consistent with CLL, or differ in IGHV-mutation status or IG isotype from the CLL counterpart, thereby retaining tolerance constraints. The precision of our analysis at this level, however, is not sufficient to assert this with complete confidence. Thus, in patients with CLL, the effectiveness of receptor editing and GC reaction checkpoints might be reduced, allowing putatively dangerous H-L CLL pairing to occur and to be recruited into a GC response, where they can differentiate into antigen-experienced cells with or without accumulation of specific SHMs and isotypes.

## Data availability statement

The data presented in the study are deposited in the SRA repository, accession number PRJNA931941 and PRJNA381394.

## Ethics statement

The studies involving human participants were reviewed and approved by Institutional Review Board of Northwell Health. The patients/participants provided their written informed consent to participate in this study.

## Author contributions

SV, DB, and NC conceived the study. SV performed experiments and analyzed data. AA and KS carried out bioinformatic analyses. AN and GF performed the single cell IGHV-IGHD-IGHJ sequencing. DB, FP and AM helped with the experiments. X-JY and SY participated in manuscript preparation. KR and JB provided patient samples and clinical correlations NC directed the experiments. SV and NC wrote the initial and final versions of the manuscript. All authors contributed to the article and approved the submitted version.
