# Supplementary figures and images for: CLL stereotyped B-cell receptor immunoglobulin sequences are recurrent in the B-cell repertoire of healthy individuals: Apparent lack of central and early peripheral tolerance censoring
Source: Front Oncol. 2023 Mar 17;13:1112879. doi: 10.3389/fonc.2023.1112879 (PMC10063922; doi:10.3389/fonc.2023.1112879)

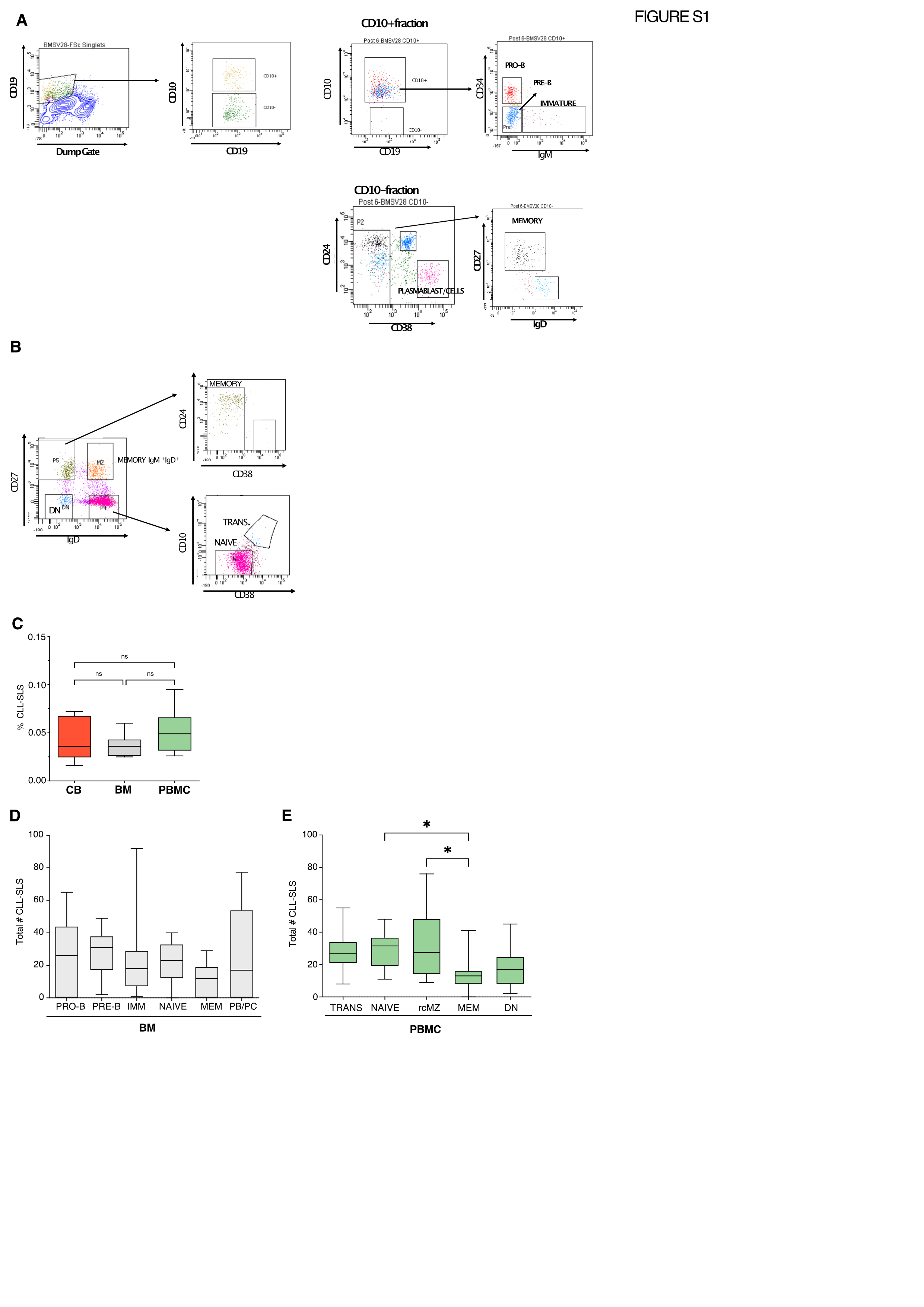

Supplement: Supplementary file 1 [file Image_1.jpeg]

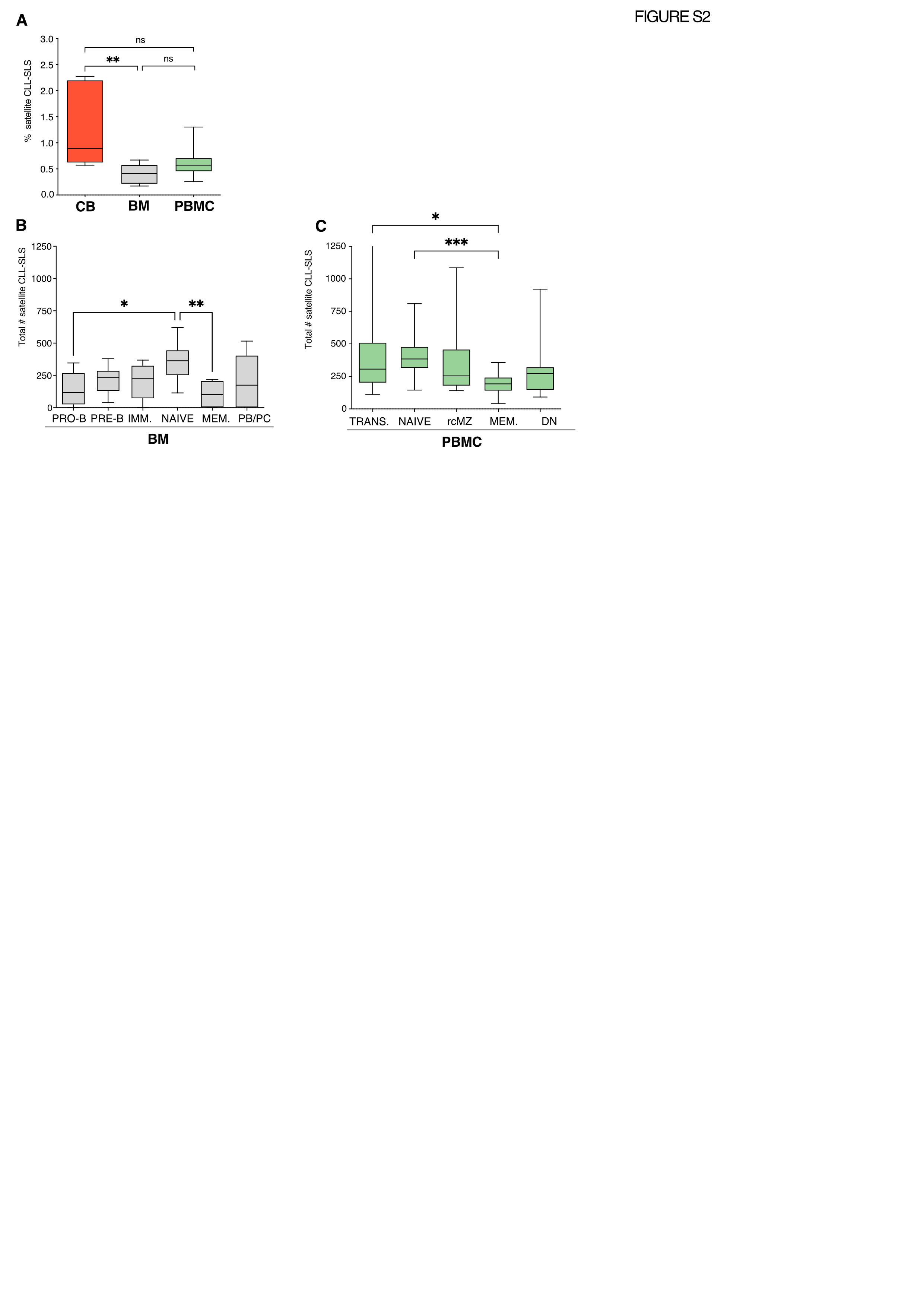

Supplement: Supplementary file 2 [file Image_2.jpeg]

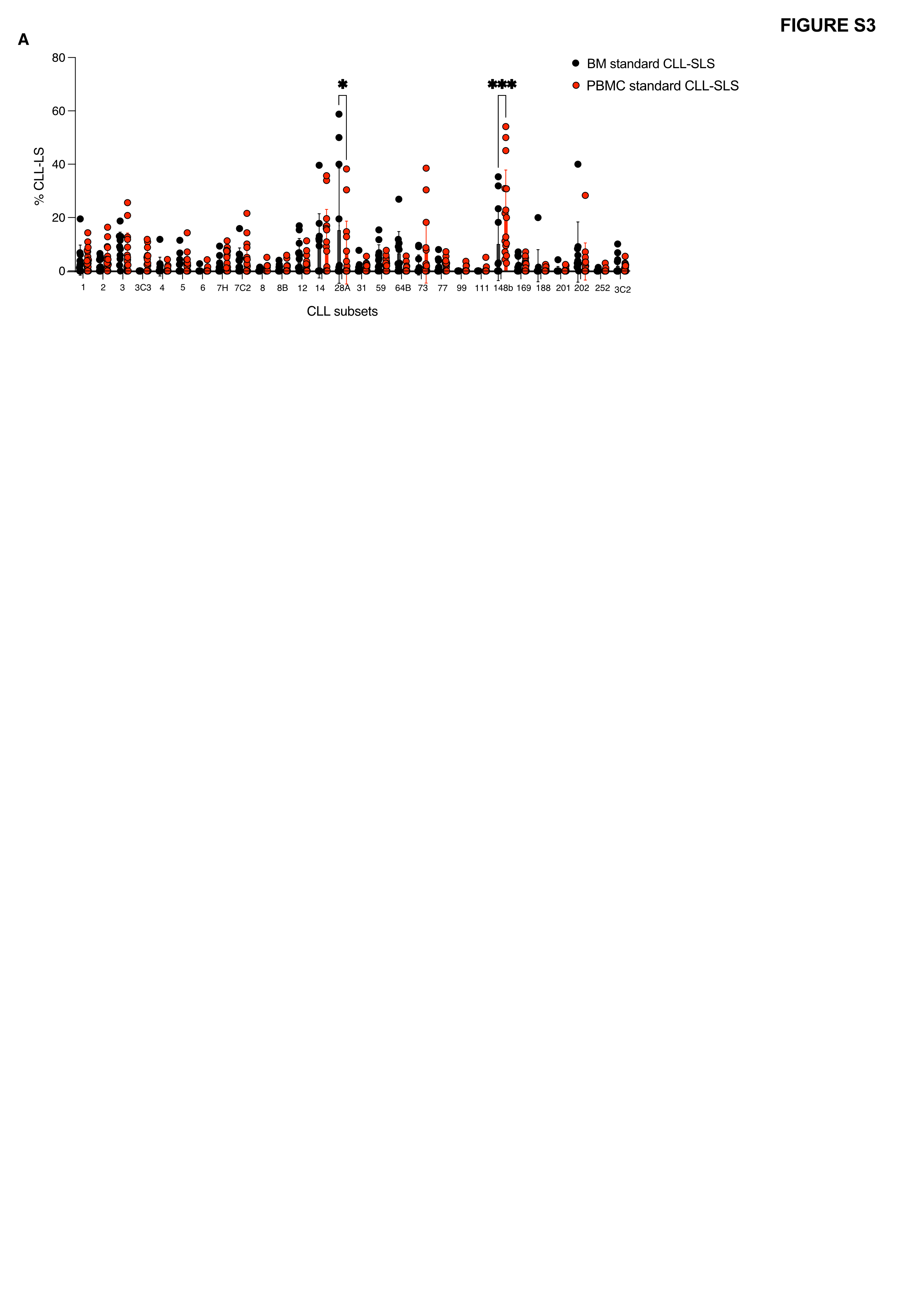

Supplement: Supplementary file 3 [file Image_3.jpeg]

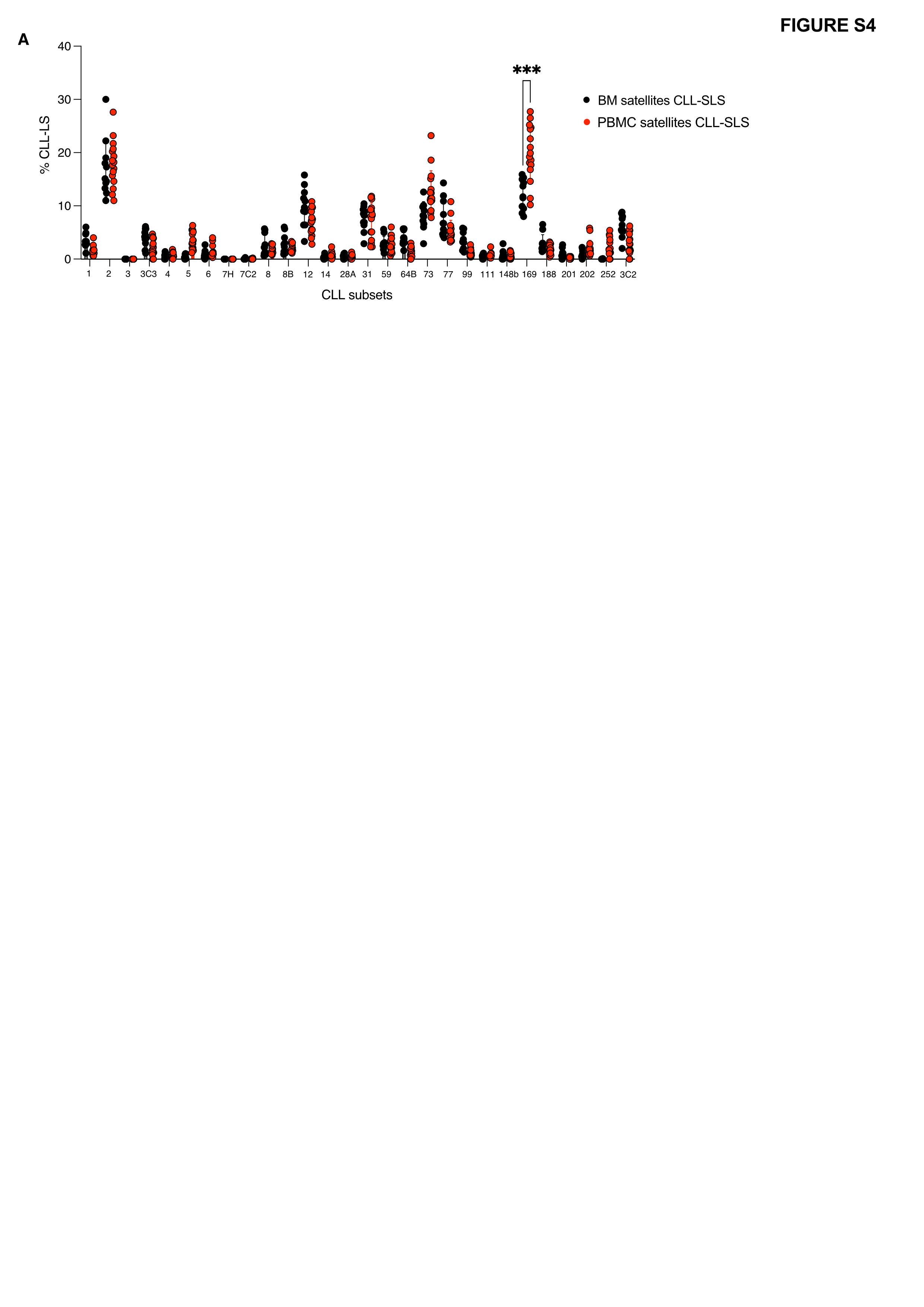

Supplement: Supplementary file 4 [file Image_4.jpeg]

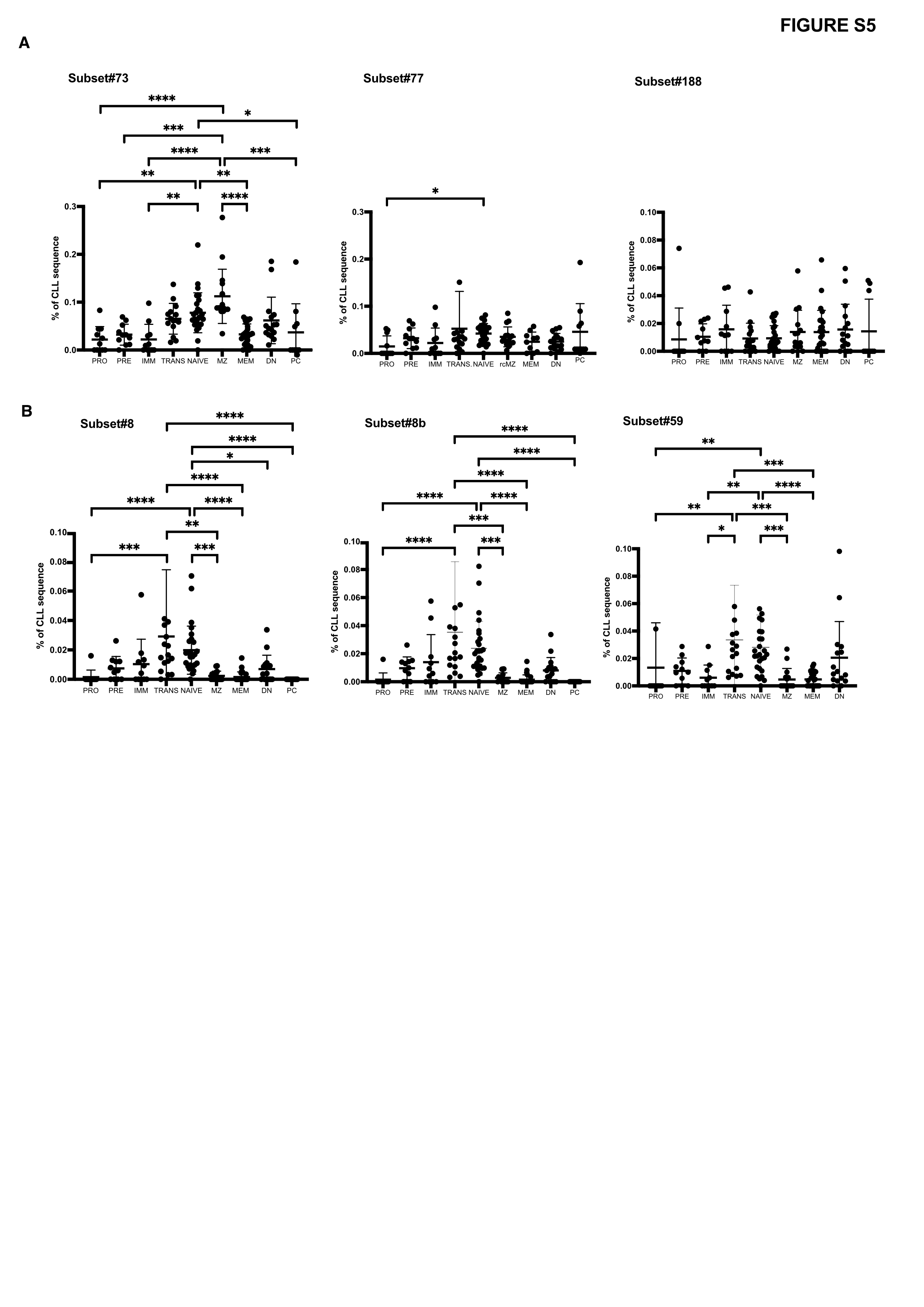

Supplement: Supplementary file 5 [file Image_5.jpeg]

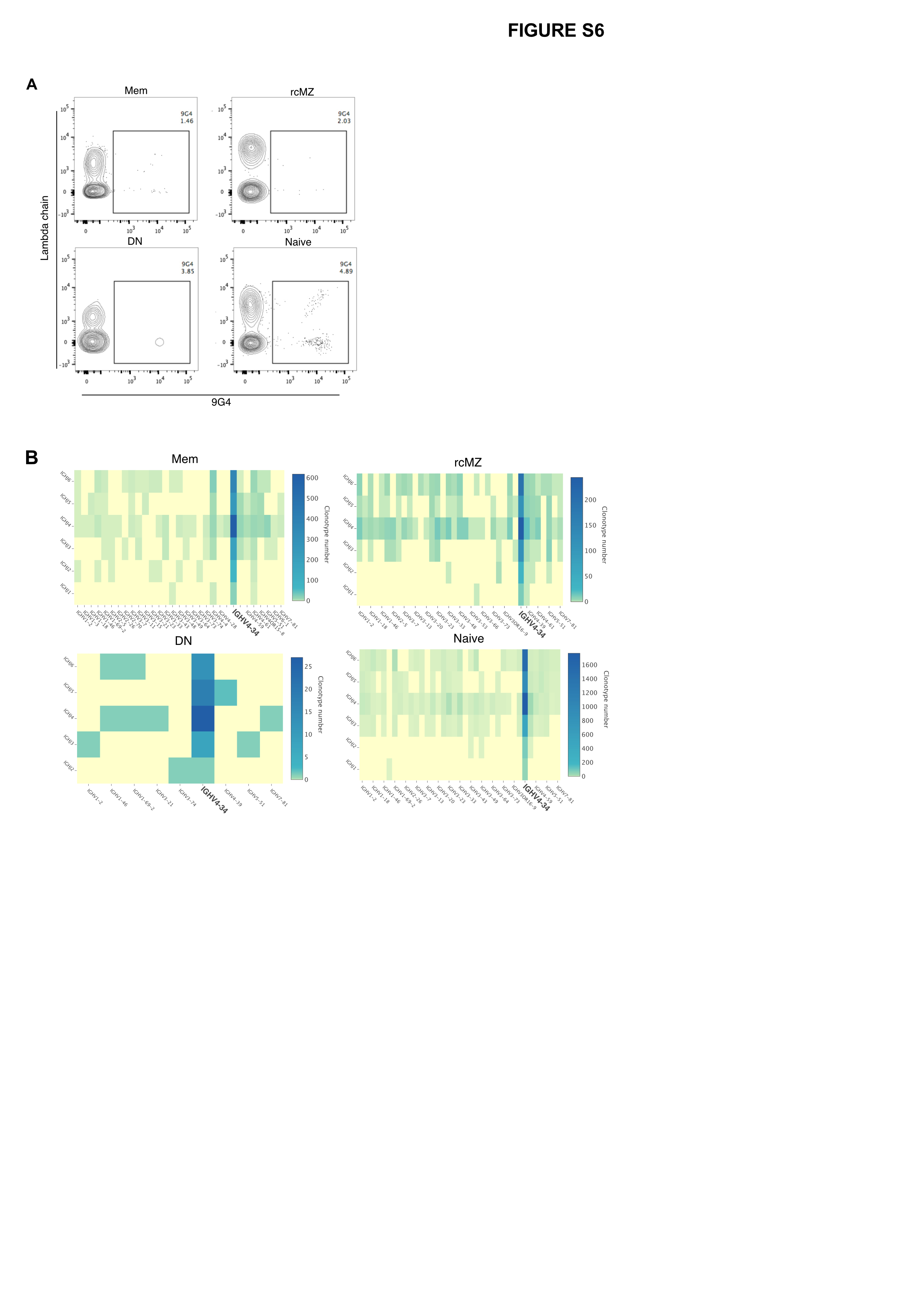

Supplement: Supplementary file 6 [file Image_6.jpeg]
